# Cancer care in times of conflict: cross border care in Pakistan of patients from Afghanistan

**DOI:** 10.3332/ecancer.2020.1018

**Published:** 2020-03-05

**Authors:** Muhammed Aasim Yusuf, Shoaib Fahad Hussain, Faisal Sultan, Farhana Badar, Richard Sullivan

**Affiliations:** 1 Shaukat Khanum Memorial Cancer Hospital and Research Centre, Lahore, Pakistan; 2 Conflict and Health Research Group, Institute of Cancer Policy, King’s College London, London, United Kingdom; * Joint First Authors.

**Keywords:** Afghanistan, Pakistan, cancer, migration, conflict and health, global health

## Abstract

Armed conflict in Afghanistan has continued for close to 40 years and has devastated its health infrastructure. The lack of a cancer care infrastructure has meant that many Afghans seek cancer care in neighbouring countries, like Pakistan. There remains a significant lack of empirical data on the new therapeutic geographies of cancer in contemporary conflicts.

This retrospective single centre study explores the therapeutic and clinical geographies of Afghan cancer patients who were treated at the Shaukat Khanum Memorial Cancer Hospital and Research Centre (SKMCH&RC) in Lahore, Pakistan over a 22-year-period (1995 to 2017) covering major periods of conflict and relative peace.

Data was available for 3,489 Afghan patients who received treatment at SKMCH&RC. The mean age at presentation was 42.7 years, and 60% were men. 30.2% came from Kabul and Nangarhar districts of Afghanistan, which have relatively short travel times to Pakistan, but patients from all parts of Afghanistan migrated to SKMCH&RC for treatment. Overall, 34.1% were diagnosed with upper gastrointestinal malignancies and 55.7% presented with late stage III/IV cancer. A wide range of treatments were provided, with 25.4% of patients receiving a combination of chemotherapy and radiation treatment. 52.7% of all patients were lost to follow-up. Outcomes were more favourable for children with cancer, 42% of whom had a complete response to therapy.

Complex migration patterns, mixed political economies (refugees, forced and unforced migrants) and models of care that must be adapted to the realities of the patients rather than notional international standards all reflect the new therapeutic geographies that long-term conflict creates. This requires significant new domestic and international (e.g., United Nations High Commissioner for Refugees) policy and practises for providing cancer care in today’s contemporary conflict ecosystems that frequently cross national borders.

## Introduction

Armed conflicts cause massive disruption including loss of life, injuries, the destruction of vital infrastructure and forced migration, with all the resulting short and long term socio-economic, political and health consequences. The world is witnessing the highest levels of human displacement due to persecution, conflict and human rights abuses in modern history with approximately 65.6 million refugees and displaced people worldwide in 2016 [1]. The international community has managed multiple refugee crises, mostly through United Nations (UN) agencies and non-governmental organisations providing acute medical care as well as controlling public health issues such as malnutrition and infectious disease outbreaks. However, non-communicable diseases (NCDs) affecting refugee and migrant populations, especially cancer, have received little attention both politically, and within the ecosystem of humanitarian medicine [2].

Migrant populations are usually unfamiliar with health systems in other countries and are often not enrolled in formal healthcare programmes [3, 4]. In some countries, such as Lebanon, refugees only receive secondary or tertiary care if funded by the United Nations High Commissioner for Refugees (UNHCR); with an 83% funding deficit, the UNHCR can only afford to fund few cancer cases [5]. Globally, most of the burden for caring for migrant populations including refugees with cancer falls on host countries and on out-of-pocket payments.

Pakistan shares a long, and in places, still disputed border with Afghanistan. Following the invasion of Afghanistan by the Union of Soviet Socialist Republics in 1979, millions of people from Afghanistan crossed this border to seek refuge in Pakistan [1]. At its peak, the refugee population within Pakistan numbered over 3.3 million people [1]. The continuation of conflict within Afghanistan since 2001 coupled to the poor healthcare infrastructure has meant that Afghan migrants continue to cross borders to present within Pakistan along ill-defined and complex therapeutic geographies, as seen in other conflict-affected countries [6]. In addition to a variety of smaller public and many private hospitals, Afghan migrants increasingly present to major apex cancer centres such as the Shaukat Khanum Memorial Cancer Hospital and Research Centre, Lahore.

**PANEL A: PATIENT SELECTION CRITERIA**

SKMCH&RC accepts patients based on diagnosis and cancer stage with an emphasis on accepting those most likely to be curable.

**PANEL B: SUPPORTING AFGHAN PATIENTS**

**ACCOMMODATION**

Most chemotherapy and radiation treatments provided at SKMCH&RC are elective/outpatient procedures requiring many patients to seek temporary accommodation near one of our hospitals.

Many patients, including Afghan patients, are unable to afford private accommodation, leading to the emergence of SKMCH&RC-affiliated hostel facilities which provide free accommodation and meals as well as individual carer support, for up to 300 patients.

For geographical reasons, staff and many patients who access these facilities speak Pashto and Farsi which helps provide informal psychosocial support and may help ameliorate the difficulties these patients face.

**WALK-IN CLINICS**

SKMCH&RC also operates a network of walk-in clinics all over Pakistan which patients with a suspected or established cancer diagnosis can attend for further management.

There are an estimated 170,000 to 200,000 new cancer diagnoses every year in Pakistan. In 2017 almost 45,000 new cancer patients attended one of our walk-in clinics seeking treatment.

Nearly 30% of all patients seen in the hospital in Lahore come from Khyber Pakhtunkhwa, the adjoining tribal areas of Pakistan, and from Afghanistan. Yet little is known about the clinical, geographic and socio-demographic features of migrants of Afghan origin. Here, we present a retrospective unselected hospital registry analysis of these patients to understand and illuminate the complex socio-demographics, geography and care of such migrant populations seeking care in a host country.

## Methods

SKMCH&RC uses a custom-built electronic medical record system which includes modules for patient registration, clinical information, order entry and viewing of results, as well as critical alerts.

Since 2000, all patient data has been entered directly into this system, with paper charts prior to this having been scanned and archived into the system [7]. Data of patients registered between 1st December 1995 and 1st December 2017 were collected to identify cancer patients over a 22-year period who were either identified as Afghan nationals or provided an Afghan address at the time of initial registration.

A retrospective review was performed by two independent reviewers, looking at patient demographics, including address within Afghanistan, sex, cancer diagnosis, stage, treatment provided and follow-up data, where available.

## Results

A total of 4,039 patients were identified as Afghan nationals, i.e., having provided an Afghan address at the time of initial registration between 1995 and 2017. This represents 4.84% of all new patients (*n* = 83,477) seen at SKMCH&RC [8]. 550 of these patients did not receive any further care at SKMCH&RC for various reasons including the diagnosis of benign disease and not returning for follow-up after being requested to obtain further diagnostic investigations. Final analysis is based on the remaining 3,489 patients of which 87.4% had an address in Afghanistan (Table A1).

**Figure 1.**
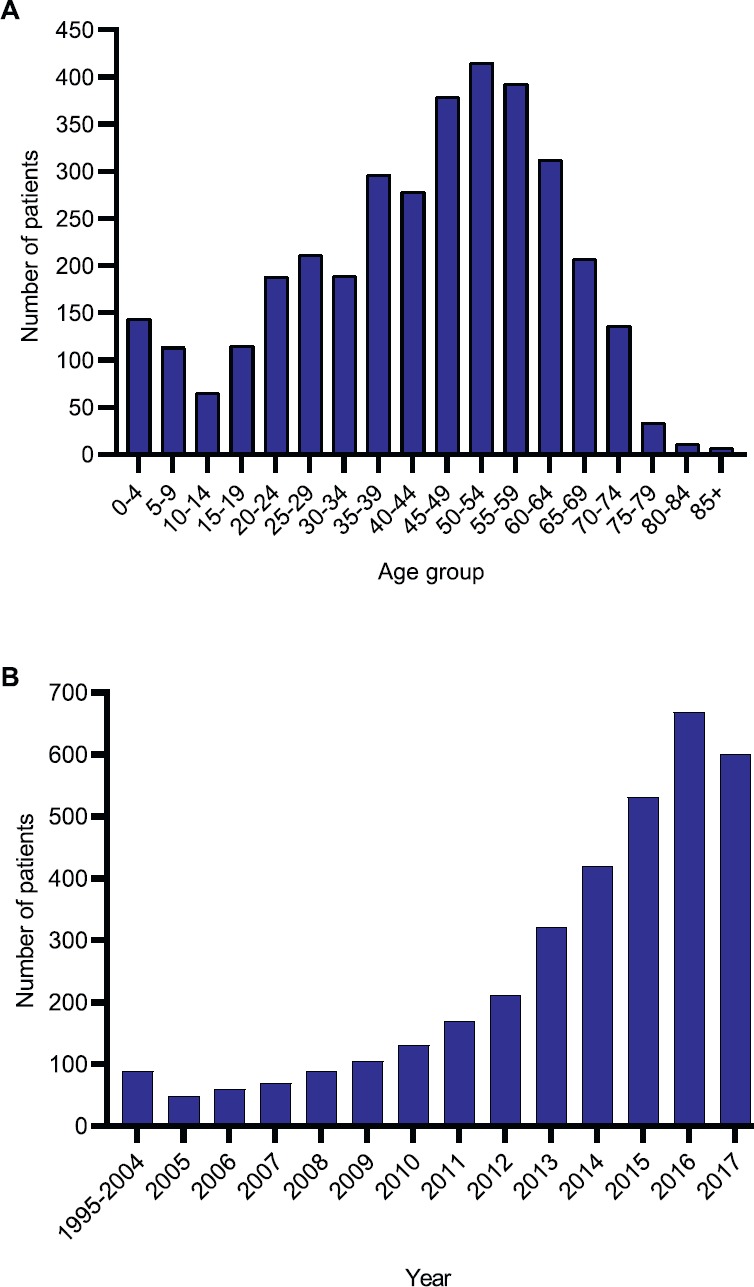
Afghan migrants treated at SKMCH&RC (n = 3,489) from 1995 to 2017. (A) Age distribution. (B) Frequency of Afghan patients by year.

The mean age at presentation was 42.7 years and median age was 45 years. 60% of patients were male and 12% were children aged ≤ 19-year old (Figure 1A). There was a rapid increase in the number of Afghan migrants presenting from 2008 onwards, coinciding with a major increase in conflict in Afghanistan (Figure 1B).

**Figure 2.**
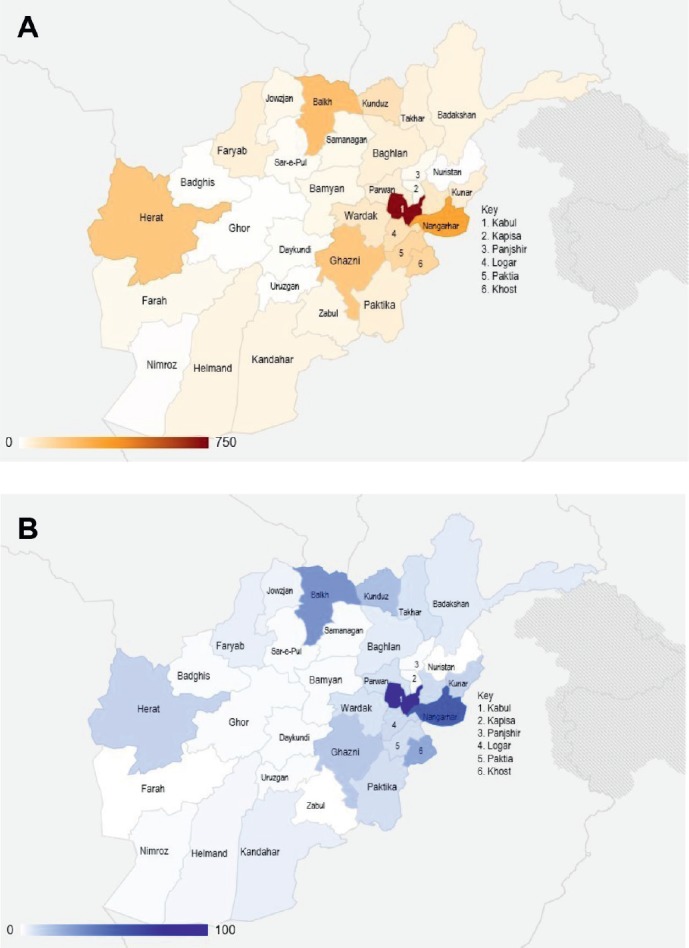
Distribution of patients by province of origin in Afghanistan. (A) Overall. (B) Paediatric only.

The largest number came from Kabul (20.9%) followed by Nangarhar (9.3%), Balkh (6.2%), Ghazni (5.3%) and Herat (5.3%) (Figure 2, Table A6). The province of origin for 19.3% of patients was unknown. The largest number of paediatric patients also came from Kabul (21.2%) followed by Nangarhar (12.7%), Balkh (7.4%) and Khost (5.8%). The province of origin for 19.2% of paediatric patients was unknown (Figure 2, Table A6). Data on distribution by gender has been provided in Table A7.

Driving times between Lahore, Pakistan and various provinces of Afghanistan from which the majority of Afghan migrants originated were estimated (from Google Maps^™^) were calculated as follows: 11–15 hours for Kabul; 8–13 hours for Nangarhar; 17 hours for Balkh; 13–14 hours for Ghazni and 23–26 hours for Herat. Each of the other provinces together accounted for less than 5% of the patients.

**Figure 3.**
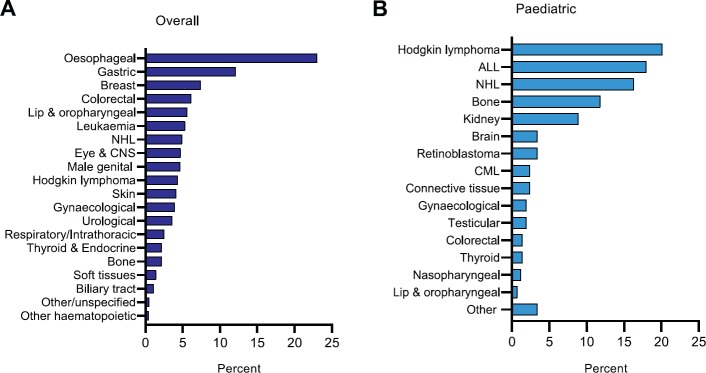
Distribution by site. (A) Overall (B) Paediatric. ALL: acute lymphoblastic leukaemia; CML: chronic myeloid leukaemia; CNS: central nervous system; NHL: non-Hodgkin lymphoma.

Upper gastrointestinal malignancies, including oesophageal (23%) and gastric cancer (12.1%), were the most common cancers (*n* = 3,489), followed by breast (7%) and colorectal cancer (6%) (Figure 3A). Haematological malignancies, including Hodgkin lymphoma (20.1%), acute lymphoblastic leukaemia (18%) and non-Hodgkin lymphoma (16.3%), were the most common cancers in paediatric cases (*n* = 417) (Figure 3B).

Further analysis of adult patients revealed that 42% of male patients and 36.8% of female patients presented with Upper GI malignancies. 20.1% of female patients presented with breast cancer. Male genital, gynaecological, lower GI and oropharyngeal malignancies were the next most common cancers among adult males and females (Table 1a).

As discussed above, haematological malignancies were the most common cancers affecting paediatric patients. Hodgkin lymphoma (23.8%) was the most common cancer affecting male children, whereas acute lymphoblastic leukaemia (17.1%) was the most common cancer affecting female children. Bone cancer, renal and urinary system and eye and central nervous system malignancies were the next most common cancers affecting male children whereas bone, renal and urinary system and gynaecological malignancies were the next most common cancers affecting female children (Table 1b).

55.7% of all patients (*n* = 3,489) presented with advanced disease (Stage III and IV) and in 17.4%, cancer stage was not documented. Further analysis of the cohort by age group revealed that 58% of adults and 39.1% of paediatric patients presented with advanced disease. Cancer stage for 14.7% of adults and 37.2% of paediatric patients was not documented.

Overall, 52.7% of patients, including 53.9% of adults and 43.2% of paediatric patients, were lost to follow-up likely due to travel requirements. 30.4% of adult and 29.5% of paediatric patients were being actively followed up. 6.9% of adults and 15.1% of paediatric patients had died during the study period. Further analysis for cancer stage and patient outcomes by gender is presented in Table A8.

**Figure 4.**
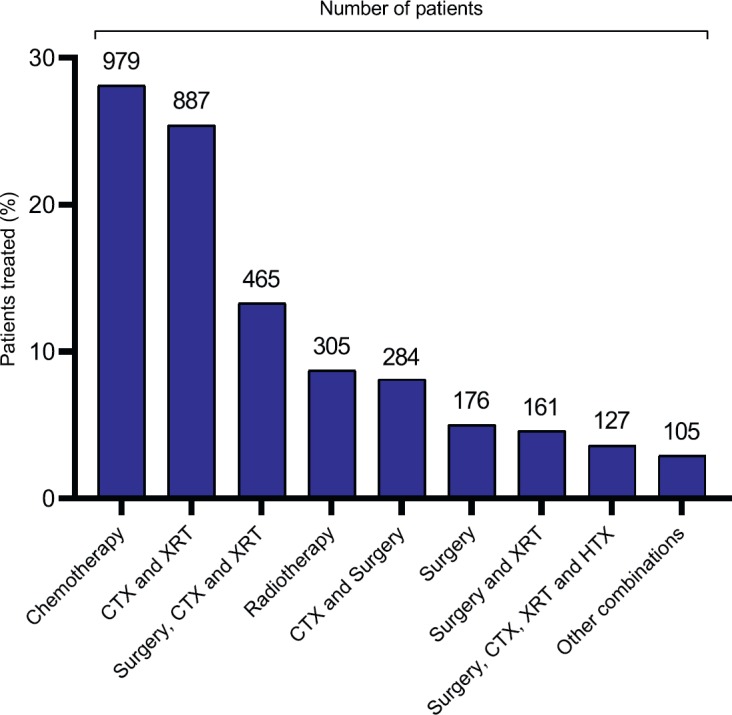
Distribution by treatment strategy.

**Figure A1.**
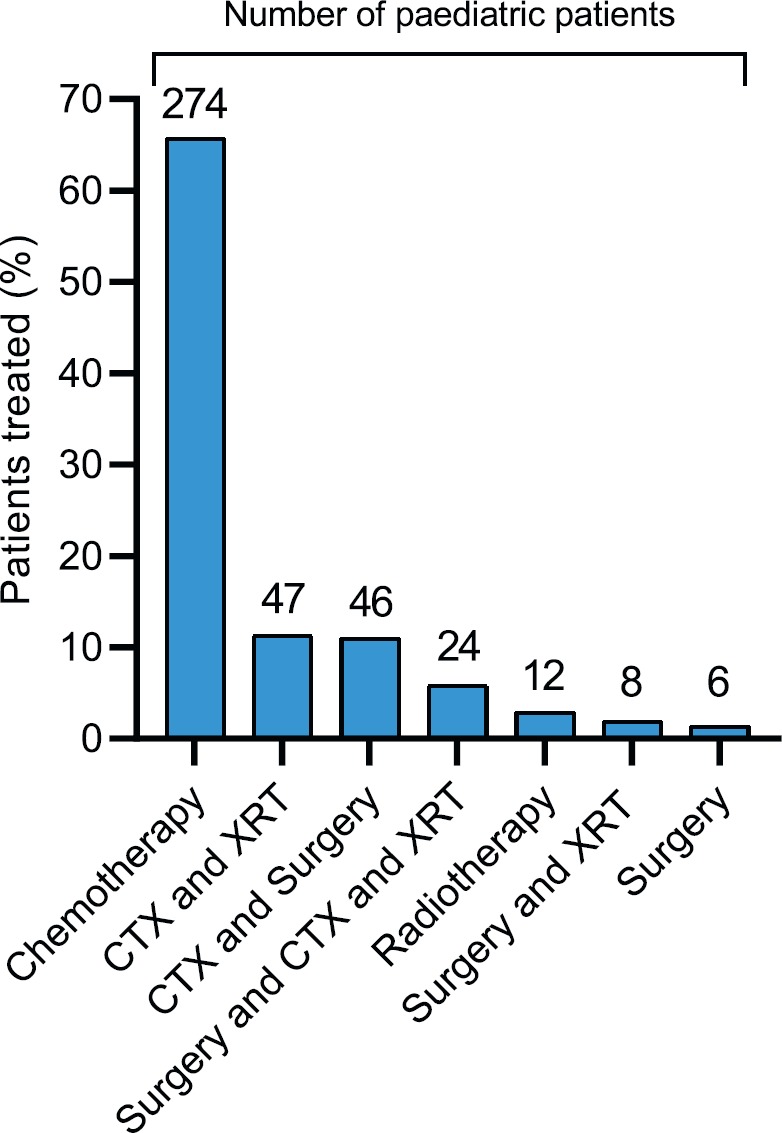
Distribution by treatment strategy in paediatric patients.

Patients received a range of treatments in keeping with the heterogeneity of cancer diagnoses (Figure 4, Table 3a). More than two-thirds of patients were treated with chemotherapy or a combination of chemotherapy (CTX) and radiotherapy (XRT). Other treatment options included surgery, hormone therapy (HTX) and immunotherapy. 33.2% of the overall study cohort achieved a complete response, 10.8% partially responded to therapy, 7.8% had stable disease and 27.8% relapsed or had progressive disease (Table A2). We were unable to assess disease outcome in 20.3% of patients. 42% of paediatric patients achieved a complete response to therapy, 10.3% partially responded, 4.6% had stable disease and 17.3% relapsed or had progressive disease. We were unable to assess disease outcome in 25.9% of paediatric patients (see Tables A3 and A4 for treatment outcomes for male and female adult patients, respectively, and Table A5 for the paediatric migrant population). More than two-thirds of paediatric patients were treated with CTX alone, in keeping with the nature of cancer diagnoses (Figure A1, Table 3b).

## Discussion

NCDs, which include the spectrum of cancers, are predicted to constitute over 80% of the global burden of disease by 2020 and will disproportionately affect Low- and Middle-Income Countries, especially refugee populations, with damaging long-term health and socio-economic consequences [9–11]. This is particularly relevant for conflict-affected regions which face a triple burden of disease encompassing communicable diseases, NCDs and trauma with limited healthcare resources and constant insecurity [2, 12, 13].

This study provides a basic demographic and clinical profile for Afghan cancer patients who were treated between 1995 and 2017 at SKMCH&RC. The majority of patients presenting to our institution from Afghanistan were treatment naïve. Most patients underwent initial investigations in Afghanistan, including a biopsy, to establish a cancer diagnosis. Although some patients underwent surgical treatment, we rarely encountered patients who had received chemotherapy, and there are currently no functional radiotherapy facilities in Afghanistan.

A significant proportion of Afghan patients presented at an advanced stage of disease. The most common cancers among adult patients in our cohort were upper gastrointestinal, breast and colorectal malignancies, whereas most paediatric patients presented with haematological malignancies (Figure 3). These findings are similar to previous studies investigating cancer among Afghan refugees in Iran and Pakistan [10, 11, 14]. The aetiology of cancer is complex but risk factors, including environmental exposure to munitions and the toxic remnants of war, poor dietary and lifestyle habits, including the heavy use of tobacco-based products such as *naswar*, and the lack of cancer surveillance programmes likely contribute to the development of cancer in this population [11, 15–18].

Over forty years of conflict has decimated Afghanistan’s health infrastructure and there is minimal data on the burden of disease or resource allocation in the country [19, 20]. The lack of a cancer surveillance programme, the cost of treatment, as well as lack of knowledge about cancer treatments lead to significant delays in patients seeking medical attention and presenting with advanced disease. The situation is exacerbated by on-going conflict and insecurity, financial hardship and limited access to follow-up or definitive treatment [11].

Our data shows that there was a steep rise in the number of Afghan patients presenting to SKMCH&RC in the early 2000s. It is plausible that due to a period of relative peace in the mid-to-late 1990s fewer Afghan patients presented to Pakistan for cancer treatment. In addition, border controls at the Afghanistan-Pakistan border were virtually non-existent and Afghan nationals were able to buy and rent property in Pakistan with fewer restrictions.

There are clearly large numbers of patients needing cancer treatment within Afghanistan, many of whom are currently forced to travel long distances to seek treatment elsewhere. The journey is often dangerous and arduous, given the on-going conflict in various parts of Afghanistan. Patients often travel with several relatives, adding to overall cost as well as causing significant disruption to family life at home. Pakistan has also implemented stricter border controls since 2016 and Afghan patients are now universally required to obtain a visa to enter Pakistan which has added to the difficulties facing these patients. Most visas are issued for periods of 2–4 weeks at a time, and patients undergoing prolonged treatment must shuttle back and forth to renew their visas. Consequently, many patients miss appointments for investigations, treatment, or for follow-up which may explain the high loss to follow-up in our cohort, and our inability to assess disease outcome for 20.3% of our cohort, including 25.3% of paediatric patients.

Nevertheless, there is a trend towards increased care-seeking among Afghan patients highlighted by the steady rise of Afghan patients presenting to SKMCH&RC (Figure 1b). This may reflect increased awareness of cancer among Afghans and the impact of the international presence in Afghanistan, including on-going capacity-building efforts between health organisations such as the World Health Organisation and the Afghan government [21], but it may also be because more patients in Afghanistan are aware of the services provided by SKMCH&RC, including the fact that free treatment is offered to the majority of patients (Panel A). We also note that a significant proportion of patients present from Afghan provinces that are not geographically contiguous with Pakistan, such as Balkh and Herat, which are much closer to Iran (Figure 2). Afghan patients may choose to be treated at SKMCH&RC because it allows unrestricted access to Afghan patients and provides free cancer treatment to all patients experiencing financial hardship. Anecdotally, Afghan patients report that procuring a visa to enter Iran is also significantly more difficult than for Pakistan.

Iraq and Syria face a similar situation with conflict displacing millions of people and destroying the local oncology infrastructure [2, 22]. In Syria alone, more than half of all public hospitals have been damaged or destroyed and there are no diagnostic imaging or radiation therapy facilities available, resulting in more than 45% of Syrian patients being unable to complete their treatment in Syria [23, 24]. In Iraq, which once had a robust oncology programme, hospitals face critical pharmaceutical shortages resulting in limited doses of chemotherapy per patient [24]. This has forced people to piece together cancer care across domestic and international boundaries whilst navigating personal illness, financial hardship, threats of violence as well as visa and security/checkpoint restrictions as seen in Afghanistan [22, 26].

The conflict in Afghanistan has impaired the development of its health infrastructure and the displacement of millions of people which has placed a substantial burden on host countries’ healthcare systems. As discussed above, a significant proportion of Afghan patients presented with advanced cancers, including tumours that are significantly cheaper and easier to treat if they were detected at an earlier stage.

It is imperative that all stakeholders invest in developing a robust national cancer screening and awareness programme, which is currently lacking in Afghanistan. Funding cancer care for refugees has historically been neglected with resources and funding directed towards more acute treatments. The funding deficit is compounded by the misconception that all types of cancer have poor prognoses. Several commentators have advocated using national cancer registries to achieve better surveillance and allow forecasting of crises so that aid from international organisations can be requested beforehand. Public awareness, including information on when and where to seek help, should be made available to help detect and treat early stage cancer which is less costly and carries better prognosis [4, 27]. In addition to increasing funding for secondary and tertiary care, primary prevention and cancer surveillance strategies incorporating mobile health clinics and civil society organisations with a model akin to the Sendai Framework for Disaster Risk Reduction should be considered [4]. Afghanistan can also benefit from initiatives aimed at improving cancer control in the Muslim world such as the Programme of Action for Cancer Therapy, a collaborative effort between the International Atomic Energy Agency, the Organisation of Islamic Cooperation (OIC) and the Islamic Development Bank to bolster radiation medicine facilities, provide technical support and improve cancer care infrastructure among the 57 member states of the OIC [28].

## Limitations

This study has some important limitations. The use of paper records before 2000, as well as relatively limited data being requested from patients in earlier years, may have resulted in a number of Afghan patients being treated without being identified. Less stringent border controls before 2016 enabled many Afghan families to live in Pakistan in a state of semi-permanent residence without being formally registered or documented, and Afghan patients were able to enter and leave Pakistan with relative ease. As a result, the actual numbers of Afghan patients treated at SKMCH&RC may have been underestimated. Finally, we have used the provision of an Afghan address as a permanent place of residence as a surrogate for Afghan nationality. While there is no way of knowing whether this is accurate, we do not feel that this is likely to be a source of major discrepancy in the numbers we have provided.

## Conclusions

**PANEL C: ESTABLISHING A CANCER CENTRE IN AFGHANISTAN**

SKMCH&RC is currently engaged in discussions with the Government of Afghanistan to help to establish a national cancer centre in Kabul. We will be focusing our efforts on:

- Training Afghan physicians, nurses and allied healthcare professionals.
- Establishing a population-based cancer registry starting in Kabul and eventually extending to the rest of Afghanistan.

With more pressing and visible concerns such as primary care, maternal care and childhood vaccination programmes, cancer treatment often loses out. The WHO EMRO and other donor agencies should support such initiatives in the medium- to long-term to help address cancer care needs in Afghanistan.

While the exact magnitude of the cancer problem in Afghanistan is unknown, this is the largest dataset of Afghan cancer patients to date. We feel our data is important in drawing attention to the possible scale of the problem and highlights the importance of further work in understanding the aetiology and epidemiology of cancer in Afghanistan as well the establishment of cancer services in the country. We have attempted to draw attention to the problem of cancer diagnosis and treatment in Afghanistan and to provide some initial data as to incidence, tumour types, most common age at presentation and regional distribution in our cohort. There may also be important differences in cancer incidence, at least between Afghanistan and Pakistan, not previously noted.

Future research should concentrate on building a clearer understanding of the incidence and prevalence of cancers within Afghanistan assess the impact of dietary and lifestyle habits within the Afghan population, as well as environmental factors including the effects of munitions and the toxic remnants of war. Further work is also needed to identify barriers to cancer care-seeking among the Afghan population including cultural and societal factors to better inform cancer control policy and tailor cancer awareness campaigns in the country.

Our data is likely to be helpful for national health policy planners, as well as for international funding agencies, such as the UNHCR and others. We have also provided data on the possible distribution of cancers within this population. Our data provides important preliminary information for health care policy planners, as well as for donor agencies within Afghanistan, especially given the paucity of information currently available. The nascent efforts now underway to establish cancer services in Afghanistan, with which we are involved, will hopefully form the nucleus around which such services can be planned in the future.

## List of abbreviations

ALL: Acute lymphoblastic leukaemia
CML: Chronic myeloid leukaemia
CNS: Central nervous system
CTX: Chemotherapy
EMRO: WHO Regional Office for the Eastern Mediterranean
GI: Gastrointestinal
HTX: Hormone therapy
NCD: Non-communicable disease
NHL: Non-Hodgkin lymphoma
OIC: Organisation of Islamic Cooperation
SKCMH&RC: Shaukat Khanum Memorial Cancer Hospital and Research Centre
UN: United Nations
UNHCR: United Nations High Commissioner for Refugees
XRT: Radiotherapy

## Conflicts of interest

None to declare.

## Disclosure of results at a meeting

None.

## Institutional review

Shaukat Khanum Memorial Cancer Hospital and Research Centre Institutional Review Board Number: 07-07-17-21.

## Funding

This publication is funded through the UK Research and Innovation GCRF RESEARCH FOR HEALTH IN CONFLICT (R4HC-MENA); developing capability, partnerships and research in the Middle and Near East (MENA) ES/P010962/1.

## Figures and Tables

**Table 1a.** Distribution of adult patients by cancer type and gender.

Cancer type	No. (%)	
	Male (*n* = 1,801)	Female (*n* = 1,271)
Upper GI	757 (42.0)	468 (36.8)
Breast	2 (0.1)	255 (20.1)
Genital organs	153 (8.5)	124 (9.8)
Lower GI	145 (8.1)	61 (4.8)
Lip, oral cavity and pharynx	130 (7.2)	58 (4.6)
Skin	103 (5.7)	40 (3.1)
Eye and CNS	84 (4.7)	53 (4.2)
Non-Hodgkin lymphoma	73 (4.1)	29 (2.3)
Respiratory system and intrathoracic organs	68 (3.8)	16 (1.3)
Renal and urinary system	66 (3.7)	23 (1.8)
Hodgkin lymphoma	43 (2.4)	22 (1.7)
Chronic myeloid leukaemia	28 (1.6)	25 (2.0)
Soft tissues	23 (1.3)	17 (1.3)
Chronic lymphocytic leukaemia	22 (1.3)	3 (0.2)
Biliary tract	24 (1.3)	14 (1.1)
Thyroid and other endocrine glands	21 (1.2)	49 (3.9)
Bone	22 (1.2)	6 (0.5)
Acute lymphoblastic leukaemia	12 (0.7)	4 (0.3)
Other and unspecified	13 (0.7)	2 (0.2)
Other haematopoietic	9 (0.5)	2 (0.2)
Other leukaemia	3 (0.2)	0 (0.0)

**Table 1b.** Distribution of paediatric patients by cancer type and gender.

Cancer type	No. (%)	
	Male	Female
	(*n* = 294)	(*n* = 123)
Hodgkin lymphoma	70 (23.8)	14 (11.4)
Acute lymphoblastic leukaemia	54 (18.4)	21 (17.1)
Non-Hodgkin lymphoma	54 (18.4)	14 (11.4)
Bone	33 (11.2)	16 (13.0)
Renal and urinary system	22 (7.5)	15 (12.2)
Eye and CNS	21 (7.1)	7 (5.7)
Genital organs	8 (2.7)	11 (8.9)
Chronic myeloid leukaemia	8 (2.7)	2 (1.6)
Lip, oral cavity and pharynx	6 (2.0)	3 (2.4)
Lower GI	5 (1.7)	1 (0.8)
Thyroid and other endocrine glands	5 (1.7)	3 (2.4)
Soft tissues	2 (0.7)	8 (6.5)
Other leukaemia	3 (1.0)	1 (0.8)
Other and unspecified	2 (0.7)	2 (1.6)
Respiratory system and intrathoracic organs	1 (0.3)	1 (0.8)
Upper GI	0 (0.0)	1 (0.8)
Biliary tract	0 (0.0)	1 (0.8)
Other haematopoietic	0 (0.0)	2 (1.6)

**Table 2a.** Distribution of study cohort by cancer stage.

Cancer stage	No. (%)	
	Adults (n = 3,072)	Paediatric (n = 417)
Stage 0	6 (0.2)	0 (0.0)
Stage I	258 (8.4)	44 (10.6)
Stage II	578 (18.8)	55 (13.2)
Stage III	1172 (38.2)	86 (20.6)
Stage IV	607 (19.8)	77 (18.5)
Unknown/not applicable	451 (14.7)	155 (37.2)

**Table 2b.** Distribution of study cohort by select outcomes.

Select outcomes	No. (%)	
	Adults (*n* = 3,072)	Paediatric (*n* = 417)
Active follow-up	935 (30.4)	123 (29.5)
Active treatment	187 (6.1)	33 (7.9)
Discharged	80 (2.6)	18 (4.3)
Died	213 (6.9)	63 (15.1)

**Table 3a.** Treatment data for the most common cancers affecting adults. CTX = chemotherapy, XRT = radiotherapy, HTX = hormone therapy.

	Upper GI (*n* = 1,225)	Breast (*n* = 257)	Lower GI (*n* = 206)	Lip, oral cavity and pharynx (*n* = 188)	Male genital (*n* = 153)
Chemotherapy	308	10	47	7	47
Radiotherapy	37	5	3	36	2
Surgery	13	4	16	2	10
CTX+XRT	481	8	48	107	5
CTX+Surgery	103	21	26	1	29
XRT+Surgery	6	2	1	24	4
CTX+XRT+Surgery	276	43	62	11	4
Hormone therapy	-	1	-	-	21
HTX+CTX	-	2	-	-	3
HTX+XRT	-	-	-	-	10
HTX+Surgery	-	5	1	-	10
HTX+CTX+Surgery	-	21	-	-	1
HTX+CTX+XRT	-	14	-	-	6
HTX+CTX+XRT +Surgery	1	121	2	-	1

**Table 3b.** Treatment data for the most common cancers affecting children. CTX = chemotherapy, XRT = radiotherapy.

	Leukaemia (*n* = 89)	Hodgkin lymphoma (*n* = 84)	Non-Hodgkin lymphoma (*n* = 68)	Bone (*n* = 49)	Renal and Urinary organs (*n* = 37)
Chemotherapy	89	66	63	11	11
Radiotherapy	-	-	2	-	-
Surgery	-	-	-	1	2
CTX+XRT	-	17	2	6	7
CTX+Surgery	-		1	24	7
CTX+XRT +Surgery	-	1	-	7	10

## Appendix

**Table A1.** Distribution of study cohort by country of birth and country of residence.

Country of residence	Country of birth		
	Afghanistan	Pakistan	Total
Afghanistan	2,713	337	3,050
Pakistan	439	0	439
Total	3,152	337	3,489

**Table A2.** Distribution of study cohort by treatment response.

Treatment outcome	No. (%)	
	Overall (*n* = 3,489)	Paediatric (*n* = 417)
Complete response	1,159 (33.2)	175 (42.0)
Partial response	377 (10.8)	43 (10.3)
Stable disease	273 (7.8)	19 (4.6)
Progression/Relapse	970 (27.8)	72 (17.3)
Unknown	710 (20.3)	108 (25.9)

**Table A3.** Treatment outcomes and cancer stage for the top five cancers affecting adult male patients aged ≥ 19-year old.

Cancer diagnosis	Treatment outcome	Number of patients						
		Stage						
		0	I	II	III	IV	NK/NA	Total
Oesophageal	Complete Response	-	3	18	55	4	1	81
	Partial Response	-	1	11	63	9	1	85
	Stable disease	-	1	3	29	6	1	40
	Progression/Relapse	-	1	15	94	28	4	142
	Unknown	-	2	6	48	13	5	74
	Total	-	8	53	289	60	12	422
Stomach	Complete Response	-	3	14	31	5	0	53
	Partial Response	-	1	8	21	11	1	42
	Stable disease	-	0	10	22	14	0	46
	Progression/Relapse	-	0	26	57	35	6	124
	Unknown	-	0	7	35	22	6	70
	Total	-	4	65	166	87	13	335
Colorectal	Complete Response	-	2	18	20	0	1	41
	Partial Response	-	0	1	8	0	1	10
	Stable disease	-	0	0	6	3	0	9
	Progression/Relapse	-	1	3	37	14	1	56
	Unknown	-	1	2	16	5	1	25
	Total	-	4	24	87	22	4	141
Skin	Complete Response	1	17	19	6	3	14	60
	Partial Response	0	0	1	0	0	2	3
	Stable disease	0	0	0	1	1	0	2
	Progression/Relapse	0	3	5	4	0	7	19
	Unknown	0	2	5	4	1	7	19
	Total	1	22	30	15	5	30	103
Testicular	Complete Response	-	19	4	5	0	3	31
	Partial Response	-	1	1	12	1	0	15
	Stable disease	-	1	2	9	0	0	12
	Progression/Relapse	-	0	0	9	5	0	14
	Unknown	-	3	3	7	0	0	13
	Total	-	24	10	42	6	3	85
Total	Complete Response	1	44	73	117	12	19	266
	Partial Response	0	3	22	104	21	5	155
	Stable disease	0	2	15	67	24	1	109
	Progression/Relapse	0	5	49	201	82	18	355
	Unknown	0	8	23	110	41	19	201
	Total	1	62	182	599	180	62	1,086

NK: not known, NA: not applicable.

**Table A4.** Treatment outcomes and cancer stage for the top five cancers affecting adult female patients aged ≥ 19-year old.

Cancer diagnosis	Treatment outcome	Number of patients						
		Stage						
		0	I	II	III	IV	NK/NA	Total
Oesophageal	Complete Response	-	7	16	69	4	2	98
	Partial Response	-	0	8	44	14	0	66
	Stable disease	-	0	1	27	3	1	32
	Progression/Relapse	-	0	8	82	23	5	118
	Unknown	-	0	7	57	3	1	68
	Total	-	7	40	279	47	9	382
Breast	Complete Response	3	13	130	17	1	7	171
	Partial Response	0	0	2	0	6	1	9
	Progression/Relapse	0	0	18	8	9	1	36
	Unknown	0	3	26	5	2	3	39
	Total	3	16	176	30	18	12	255
Stomach	Complete Response	-	0	4	10	1	0	15
	Partial Response	-	0	1	7	3	1	12
	Stable disease	-	0	4	6	5	0	15
	Progression/Relapse	-	0	5	21	5	2	33
	Unknown	-	0	1	6	4	0	11
	Total	-	0	15	50	18	3	86
Cervical/Uterine	Complete Response	1	9	13	1	3	2	29
	Partial Response	0	0	6	1	3	0	10
	Stable disease	0	1	1	1	0	0	3
	Progression/Relapse	0	2	5	4	5	0	16
	Unknown	0	0	5	3	2	0	10
	Total	1	12	30	10	13	2	68
Colorectal	Complete Response	-	0	11	11	0	0	22
	Partial Response	-	0	0	2	1	0	3
	Stable disease	-	0	1	5	1	0	7
	Progression/Relapse	-	0	6	15	3	0	24
	Unknown	-	0	0	4	0	1	5
	Total	-	0	18	37	5	1	61
Total	Complete Response	4	29	174	108	9	11	335
	Partial Response	0	0	17	54	27	2	100
	Stable disease	0	1	7	39	9	1	57
	Progression/Relapse	0	2	42	130	45	8	227
	Unknown	0	3	39	75	11	5	133
	Total	4	35	279	406	101	27	852

NK: not known, NA: not applicable.

**Table A5.** Treatment outcomes and cancer stage for the top five cancers affecting paediatric patients aged <19-year old.

Cancer diagnosis	Treatment outcome	Number of patients					
		Stage					
		I	II	III	IV	NK/NA	Total
Hodgkin lymphoma	Complete Response	-	20	31	14	-	65
	Partial Response	-	5	4	3	-	12
	Stable disease	-	0	1	0	-	1
	Progression/Relapse	-	0	2	4	-	6
	Total	-	25	38	21	-	84
Acute lymphoblastic leukaemia	Complete Response	-	-	-	-	15	15
	Progression/Relapse	-	-	-	-	8	8
	Unknown	-	-	-	-	52	52
	Total	-	-	-	-	75	75
Non-Hodgkin lymphoma	Complete Response	3	8	17	3	0	31
	Partial Response	0	0	7	6	0	13
	Stable disease	0	0	0	1	0	1
	Progression/Relapse	0	0	2	2	0	4
	Unknown	0	2	5	11	1	19
	Total	3	10	31	23	1	68
Bone	Complete Response	8	1	-	3	0	12
	Partial Response	3	0	-	2	1	6
	Stable disease	1	1	-	0	2	4
	Progression/Relapse	2	5	-	6	7	20
	Unknown	2	2	-	1	2	7
	Total	16	9	-	12	12	49
Renal	Complete Response	1	2	1	2	6	12
	Partial Response	0	0	0	4	2	6
	Stable disease	0	1	0	0	1	2
	Progression/Relapse	0	0	1	4	5	10
	Unknown	1	1	0	2	3	7
	Total	2	4	2	12	17	37
Total	Complete Response	12	31	49	22	21	135
	Partial Response	3	5	11	15	3	37
	Stable disease	1	2	1	1	3	8
	Progression/Relapse	2	5	5	16	20	48
	Unknown	3	5	5	14	58	85
	Total	21	48	71	68	105	313

NK: not known, NA: not applicable.

**Table A6.** Distribution of study cohort by province of origin.

Province	No. (%)	
	Overall (*n* = 3,489)	Paediatric (*n* = 417)
Kabul	730 (20.9)	88 (21.1)
Nangarhar	325 (9.3)	53 (12.7)
Balkh	216 (6.2)	31 (7.4)
Ghazni	186 (5.3)	14 (3.4)
Herat	184 (5.3)	11 (2.6)
Khost	136 (3.9)	24 (5.8)
Paktia	130 (3.7)	8 (1.9)
Kunduz	94 (2.7)	17 (4.1)
Logar	95 (2.7)	9 (2.2)
Laghman	73 (2.1)	9 (2.2)
Wardak	74 (2.1)	7 (1.7)
Baghlan	48 (1.4)	5 (1.2)
Kunar	50 (1.4)	11 (2.6)
Paktika	49 (1.4)	8 (1.9)
Parwan	50 (1.4)	7 (1.7)
Faryab	47 (1.3)	4 (1.0)
Takhar	41 (1.2)	7 (1.7)
Badakshan	37 (1.1)	5 (1.2)
Kandahar	32 (0.9)	4 (1.0)
Helmand	32 (0.9)	-
Zabul	28 (0.8)	-
Jowzjan	25 (0.7)	-
Bamyan	22 (0.6)	-
Samangan	22 (0.6)	-
Farah	20 (0.6)	-
Panjshir	17 (0.5)	-
Kapisa	14 (0.4)	-
Sar-e-Pul	10 (0.3)	-
Daykundi	7 (0.2)	-
Nimroz	6 (0.2)	-
Badghis	4 (0.1)	-
Ghor	5 (0.1)	-
Uruzgan	5 (0.1)	-
Nuristan	2 (0.1)	-
Other (combined)	-	15 (3.2)
Unknown	673 (19.3)	80 (19.2)

**Table A7.** Distribution of study cohort by gender and province of origin.

Province	No. (%)			
	Adults (*n* = 3,072)		Paediatric (*n* = 417)	
	Male (*n* = 1,801)	Female (*n* = 1,271)	Male (*n* = 294)	Female (*n* = 123)
Kabul	326 (18.1)	316 (24.9)	62 (21.1)	26 (21.1)
Nangarhar	152 (8.4)	120 (9.4)	31 (10.5)	22 (17.9)
Balkh	108 (6.0)	77 (6.1)	22 (0.2)	9 (7.3)
Ghazni	101 (5.6)	71 (5.6)	12 (7.5)	2 (1.6)
Herat	94 (5.2)	79 (6.2)	7 (4.1)	4 (3.3)
Khost	77 (4.3)	35 (2.8)	14 (2.4)	10 (8.1)
Paktia	75 (4.2)	47 (3.7)	8 (4.8)	0 (0.0)
Kunduz	48 (2.7)	29 (2.3)	11 (3.7)	6 (4.9)
Logar	43 (2.4)	43 (3.4)	8 (2.7)	1 (0.8)
Laghman	39 (2.2)	25 (2.0)	7 (2.4)	2 (1.6)
Maidan Wardak	36 (2.0)	31 (2.4)	4 (1.4)	3 (2.4)
Baghlan	35 (1.9)	8 (0.6)	4 (1.4)	1 (0.8)
Faryab	34 (1.9)	9 (0.7)	3 (1.0)	1 (0.8)
Kunar	26 (1.4)	13 (1.0)	6 (2.0)	5 (4.1)
Parwan	25 (1.4)	18 (1.4)	2 (0.7)	5 (4.1)
Badakhshan	23 (1.3)	9 (0.7)	5 (1.7)	0 (0.0)
Paktika	22 (1.2)	19 (1.5)	7 (2.4)	1 (0.8)
Takhar	21 (1.2)	13 (1.0)	5 (1.7)	2 (1.6)
Kandahar	20 (1.1)	8 (0.6)	4 (1.4)	0 (0.0)
Zabul	19 (1.1)	9 (0.7)	0 (0.0)	0 (0.0)
Jowzjan	18 (1.0)	4 (0.3)	2 (0.7)	1 (0.8)
Helmand	16 (0.9)	14 (1.1)	1 (0.3)	1 (0.8)
Samangan	13 (0.6)	8 (0.6)	1 (0.3)	0 (0.2)
Bamyan	11 (0.6)	10 (0.8)	1 (0.3)	0 (0.0)
Panjshir	11 (0.6)	5 (0.4)	1 (0.3)	0 (0.0)
Farah	10 (0.6)	10 (0.8)	0 (0.0)	0 (0.0)
Kapisa	7 (0.4)	6 (0.5)	1 (0.3)	0 (0.0)
Sar-e Pul	6 (0.3)	3 (0.2)	1 (0.3)	0 (0.0)
Daykundi	5 (0.3)	1 (0.1)	1 (0.3)	0 (0.0)
Ghor	4 (0.2)	0 (0.0)	1 (0.3)	0 (0.0)
Badghis	3 (0.2)	0 (0.0)	0 (0.0)	1 (0.8)
Nimruz	3 (0.2)	2 (0.2)	1 (0.3)	0 (0.0)
Uruzgan	3 (0.2)	1 (0.1)	0 (0.0)	0 (0.0)
Nuristan	1 (0.2)	1 (0.1)	1 (0.3)	0 (0.0)
Unknown	366 (20.3)	227 (17.9)	60 (20.4)	20 (16.3)

**Table A8.** Distribution of study cohort by cancer stage and outcomes.

Characteristic	No. (%)			
	Adults (*n* = 3,072)		Paediatric (*n* = 417)	
	Male (*n* = 1,801)	Female (*n* = 1,271)	Male (*n* = 294)	Female (*n* = 123)
Cancer stage				
Stage 0	1 (0.1)	5 (0.4)	0 (0.0)	0 (0.0)
Stage I	140 (7.8)	118 (9.3)	29 (9.9)	15 (12.2)
Stage II	246 (13.7)	332 (26.1)	39 (13.3)	16 (13.0)
Stage III	704 (39.1)	468 (36.8)	68 (23.1)	18 (14.6)
Stage IV	428 (23.8)	179 (14.1)	53 (18.0)	24 (19.5)
Unknown/Not Applicable	282 (15.7)	169 (13.3)	105 (35.7)	50 (40.7)
Patient outcome				
Lost to follow-up	1,029 (57.1)	628 (49.4)	125 (42.5)	55 (44.7)
Active follow-up	483 (26.8)	452 (35.6)	85 (28.9)	38 (30.9)
Active treatment	98 (5.4)	89 (7.0)	27 (9.2)	6 (4.9)
Discharged	48 (2.7)	32 (2.5)	16 (5.4)	2 (1.6)
Died	143 (7.9)	70 (5.5)	41 (13.9)	22 (17.9)
